# Supplementary material for: Complexome profiling of the Chlamydomonas psb28 mutant reveals TEF5 as an early PSII assembly factor
Source: Plant Cell. 2025 Mar 17;37(6):koaf055. doi: 10.1093/plcell/koaf055 (PMC12164590; doi:10.1093/plcell/koaf055)
Supplement: koaf055_Supplementary_Data [file koaf055_supplementary_data.zip › psb28_interactive.zip!/plots/intensity/qualityPlots/peptides/TRX25_Cre10.g456250.t1.2.html]

### Cre10.g456250.t1.2

Trivial name: TRX25  
SeqCoverage [median]: 7.50  
Mass: 23.23 kDa  
  
 

| Sequence | MissedCleavage | Mass [Da] | wtIntensity | muIntensity | Score |
| --- | --- | --- | --- | --- | --- |
| KMEALR | 1 | 746.41 | 16391.69 | 0.00 | 131.65 |
| NLDFIARAEK | 1 | 1175.63 | 0.00 | 1575.52 | 53.60 |
|  |  |  | 16391.7 | 1575.5 |  |
